# *SNCA* Rep1 microsatellite length influences non-motor symptoms in early Parkinson’s disease

**DOI:** 10.18632/aging.104111

**Published:** 2020-10-20

**Authors:** Alisa CW Yong, Yi Jayne Tan, Yi Zhao, Zhonghao Lu, Ebonne YL Ng, Samuel YE Ng, Nicole SY Chia, Xinyi Choi, Dede Heng, Shermyn Neo, Zheyu Xu, Kay Yaw Tay, Wing Lok Au, Eng-King Tan, Louis CS Tan, Adeline SL Ng

**Affiliations:** 1 Department of Neurology, National Neuroscience Institute, Tan Tock Seng Hospital, Singapore, Singapore; 2 Department of Clinical Translational Research, Singapore General Hospital, Singapore, Singapore; 3 Department of Neurology, National Neuroscience Institute, Singapore General Hospital, Singapore, Singapore; 4 Neuroscience and Behavioural Disorders Program, Duke-NUS Medical School, Singapore, Singapore

**Keywords:** Parkinson’s disease, non-motor symptoms, SNCA, Rep1, alpha-synuclein

## Abstract

Long alpha-synuclein gene *(SNCA)* promoter (Rep1) allele-carriers are linked to higher risk for Parkinson’s disease (PD) and faster motor progression. Non-motor symptoms including autonomic, neuropsychiatric, and sleep disorders are common in PD. However, the relationship between *SNCA* Rep1 microsatellite lengths and non-motor symptoms in early PD remains to be elucidated. 171 consecutive early PD patients were recruited from tertiary clinics and genotyped for Rep1. Multivariable regression analyses were performed to examine associations between Rep1 alleles and non-motor outcome scores. Longer Rep1 alleles significantly associated with higher total Non-Motor Symptom Scale (NMSS) scores (*p*=.006) and Hospital Anxiety and Depression Scale (HADS) depression subscale scores (*p*=.002), after adjusting for covariates and Bonferroni correction. We demonstrated that *SNCA* Rep1 allele length influences overall non-motor symptom burden and depression in early PD patients. Further functional studies to evaluate the role of Rep1 in non-dopaminergic systems may unravel new therapeutic targets for non-motor symptoms in PD.

## INTRODUCTION

Parkinson’s disease (PD) is a clinically heterogenous neurodegenerative disorder associated with motor deficits and a spectrum of non-motor symptoms (NMS), including autonomic dysfunction, constipation, hyposmia, neuropsychiatric symptoms (cognitive impairment, depression and anxiety), and rapid eye movement (REM) sleep disorders. NMS can occur years before motor symptom onset, and are common throughout the disease [1], significantly impairing quality of life even at the earliest stages of PD [2]. To allow for risk stratification and early intervention, there remains a crucial need to identify patients at a higher risk of developing non-motor symptoms.

Pathologically, non-motor symptoms in PD have been linked to widespread alpha-synuclein (α-Syn) pathology beyond the dopaminergic nigrostriatal system [3], with α-Syn aggregates in Lewy bodies present in multiple areas of the central, autonomic and peripheral nervous systems as well as visceral organs [4]. This systemic distribution of α-Syn, along with dysfunction of extranigral neuronal populations, is suggested to be the neuropathological basis of most non-motor symptoms [5]. Furthermore, increased α-Syn pathological burden has been found in familial PD patients possessing multiplications of the α-Syn gene (*SNCA*) [6]; these patients have early disease onset and rapidly progress to develop significant non-motor complications including dementia and psychiatric disturbances [7].

*SNCA* expression is regulated by non-amyloid component (NACP) Rep1 (GenBank DS3481), a polymorphic dinucleotide repeat sequence located ~10 kilobases upstream of the *SNCA* transcription start site. Rep1 functions as a negative modulator of *SNCA* transcription, with variation in allele length resulting in differences in the transcriptional regulation of *SNCA* and consequently, altered α-Syn expression [8]. Pathologically, PD carriers of the shorter genotype (259/259 base pairs [bp]) display lower levels of α-Syn in blood and post-mortem brain tissue compared to those carrying longer genotypes [9]. Expanded Rep1 alleles have also been reported in multiple cohorts to confer a higher risk for sporadic PD through increased transcription of *SNCA* [10–12], potentially mimicking *SNCA* locus multiplication [13]. A large global study found that the longer 263bp allele was associated with greater risk for PD, while the shorter 259bp allele was associated with a reduced risk for the disease [14]. Additionally, 263bp carriers have shown a four-fold increased risk of faster motor decline [15]. The strong biological link is further supported by studies that demonstrated that longer Rep1 allele carriers were more cognitively impaired [16] and at greater risk for dementia [17] than carriers of the shorter allele. No study, however, has yet investigated the relationship between Rep1 length and non-motor symptoms in PD. As non-motor symptoms usually do not occur in isolation and are not mutually exclusive, determining the association between the overall non-motor symptom burden and Rep1 may unravel new insights into the role of α-Syn in NMS pathophysiology.

To address this gap in knowledge, we investigated the relationship between Rep1 allele length and NMS in a cohort of early PD patients. We hypothesised that longer Rep1 carriers will have a greater burden of non-motor symptoms compared to short allele carriers.

## RESULTS

One hundred and seventy-one early PD patients completed baseline assessments and were included in this cross-sectional study. They were stratified into short (n = 76) and long (n = 95) Rep1 carriers. Demographic and clinical features are summarized in Table 1. Mean age of the short Rep1 carrier group (63.6 ± 9.9 years) was not significantly different from the long Rep1 carrier group (64.6 ± 7.9 years) (*p* = .927). Disease duration and motor symptom severity also did not differ significantly between groups (disease duration – Rep1-Short:1.07 ± 0.8 years, Rep1-Long:1.09 ± 0.6 years, *p* = .334) (MDS-UPDRS Part III – Rep1-Short: 20.8 ± 7.9, Rep1-Long: 21.7 ± 10.7, *p* = .960). *APOE4* status was available for 154 out of 171 patients. There were 31 *APOE4* carriers (20.1%) and 123 non-carriers overall (Rep1-Short: 54 non-carriers (77.1%) and 16 carriers (22.9%); Rep1-Long: 69 non-carriers (82.1%) and 15 carriers (17.9%)). No significant differences were found in non-motor assessment scores in both the overall sample and within each Rep1 group between *APOE4* carriers and non-carriers.

**Table 1:** Demographic and clinical characteristics of PD patients.

	**Rep1-Short (n = 76)**	**Rep1-Long (n = 95)**	***P* value^b^**
Age, years	63.6 (9.9)^a^	64.6 (7.9)	.927
Sex, male	43 (56.5%)	55 (57.9%)	.863
Age at onset, years	62.5 (10.1)	63.6 (8.0)	.968
Disease duration, years	1.07 (0.8)	1.09 (0.6)	.334
MDS-UPDRS Part III: Motor	20.8 (7.9)	21.7 (10.7)	.960
H&Y stage	1.78 (0.5)	1.90 (0.5)	.030
MoCA	25.0 (3.3)	25.3 (3.8)	.225
LEDD	177.8 (114.7)	166.7 (156.5)	.223
*APOE4* carriers^c^	16 (22.9%)	15 (17.9%)	.546
*Non-Motor Assessments*			
Total NMSS Score	14.3 (10.6)	21.0 (17.7)	.020
HADS Anxiety	2.2 (2.1)	2.2 (2.8)	.397
HADS Depression	2.1 (1.6)	3.1 (2.6)	.020
Apathy Scale	8.2 (5.5)	8.8 (6.6)	.767
Fatigue Severity Scale	25.6 (12.6)	29.7 (14.1)	.067
Epworth Sleepiness Scale	5.8 (3.9)	6.2 (3.4)	.563
Pittsburgh Sleep Quality Index	4.0 (3.0)	4.8 (3.3)	.108

Abbreviation: MDS-UPDRS, Movement Disorder Society-Unified Parkinson’s Disease Rating Scale; H&Y stage, Hoehn & Yahr stage; MoCA, Montreal Cognitive Assessment; LEDD, Levodopa Equivalent Daily Dose; NMSS, Non-Motor Symptoms Scale; HADS, Hospital Anxiety and Depression Scale.

^a^Continuous variables reported as mean (standard deviation); categorical variables reported as n (%)

^b^Comparisons of variables between Rep1-Short and Rep1-Long carriers using Mann-Whitney U test for continuous variables, chi-squared test for categorical variables

^c^*APOE4* status was available for 154 out of 171 patients (Rep1-Short: n = 70, Rep1-Long: n = 84)

Multivariable linear regression analyses adjusted for age, sex, disease duration, MoCA score and LEDD revealed that longer Rep1 alleles were significantly associated with higher total NMSS scores (Rep1-Short: 14.3 ± 10.6, Rep1-Long: 21.0 ± 17.7, β = 6.4, *p* = .006), as well as with higher scores on the HADS depression subscale (Rep1-Short: 2.1 ± 1.6, Rep1-Long: 3.1 ± 2.6, β = 1.1, *p* = .002), and the FSS (Rep1-Short: 25.6 ± 12.6, Rep1-Long: 29.7 ± 14.1, β = 4.4, *p* = .032) (Table 2). After adjusting for multiple tests using Bonferroni correction, positive correlations between Rep1 allele length and total NMSS as well as HADS depression subscale scores remained significant. No patient carried pathogenic mutations in *SNCA, LRRK2* (Leucine-rich repeat kinase 2)*, PRKN* (Parkin RBR E3 Ubiquitin Protein Ligase) and *PINK1* (PTEN-induced kinase 1), but there were three *GBA* (Glucocerebrosidase) L444P carriers. These results remained after excluding all 3 *GBA* L444P carriers from the analyses.

**Table 2:** Multivariable analysis on Rep1 status and non-motor symptoms in PD patients.

	***SNCA* Rep1 status, long vs short^a^**		
	**B (SE)**	**95% CI**	***P* value**
Total NMSS Score	6.4 (2.3)	1.84 – 11.04	.006*
HADS Anxiety	0.1 (0.4)	-0.64 – 0.87	.766
HADS Depression	1.1 (0.3)	0.40 – 1.72	.002*
Apathy Scale	0.6 (0.9)	-1.29 – 2.43	.544
Fatigue Severity Scale	4.4 (2.0)	0.39 – 8.48	**.032**
Epworth Sleepiness Scale	0.3 (0.6)	-0.92 – 1.47	.654
Pittsburgh Sleep Quality Index	0.8 (0.5)	-0.12 – 1.76	.087

Abbreviation: NMSS, Non-Motor Symptoms Scale; HADS, Hospital Anxiety and Depression Scale.

^b^Multiple linear regressions with non-motor assessment scores as outcome variables and Rep1 status as independent variable, adjusted for age, sex, disease duration, MoCA score and LEDD, B is the unstandardized beta coefficient, and SE standard error. Bolded figures indicate *p* < 0.05.

^*^Significant against Bonferroni corrected α value of *p* = 0.05/7 = 0.007.

## DISCUSSION

We demonstrated for the first time that *SNCA* Rep1 polymorphism is associated with non-motor burden in a cohort of patients at the early stages of PD as assessed by the NMSS - which includes autonomic symptoms (cardiovascular, gastrointestinal, urinary and sexual function), sleep dysfunction, fatigue, apathy, mood disorders, psychosis, sensory symptoms, and cognition. Multivariable regression analyses adjusted for age, sex, disease duration, MoCA score, LEDD, and corrected for multiple testing showed significant associations between longer Rep1 alleles and greater overall non-motor symptom burden as well as depressive symptoms, as assessed by total NMSS and HADS depression subscale scores respectively. We previously reported in a separate PD cohort with more advanced disease (mean disease duration of 5.2 years) that longer Rep1 allele PD carriers showed more cognitive and motor impairment than carriers of the shorter allele [16].

Interestingly, α-Syn has been implicated in psychiatric disorders, particularly the links between Rep1 polymorphism and psychiatric symptoms. Longer Rep1 alleles were more frequent in alcohol-dependent patients compared to healthy controls [18], and correlated with greater depressive symptoms in both patients with major depressive disorder (MDD) [19] and healthy individuals [20]. Depressive symptom severity in patients with MDD also correlated with increased *SNCA* mRNA levels [19].

Pathological evidence of the potential role of *SNCA* Rep1 and/or α-Syn specifically to PD non-motor symptoms have recently been elucidated in animal models. Using a transgenic mouse model with point mutation A53T, two single nucleotide polymorphisms (SNPs) (rs11931074 (G to T) and rs3857059 (A to G)) and a Rep1 polymorphism in *SNCA,* Taguchi et al [21] observed that transgenic mice exhibited hyposmia at 9 months of age, and REM sleep behaviour disorder (RBD) as early as 5 months of age. Importantly, phosphorylated α-Syn was found in brain regions seen in PD and dementia with Lewy bodies (DLB), relevant to these non-motor symptoms, such as the sublateral dorsal tegmental nucleus and olfactory bulb, consistent with the clinical findings. Additionally, mice transgenic for *SNCA* A53T and overexpressing α-Syn were also found to exhibit olfactory dysfunction, as well as significantly lower cholinergic neurons and decreased acetylcholinesterase activity in the olfactory bulb [22].

Evidence from neuroimaging studies also suggest involvement of neurotransmitter systems in the pathophysiology of depression in PD. Using [^11^C]RTI-32 as a PET marker for dopamine and noradrenaline transporter binding, reduced binding was found in the locus coeruleus and limbic regions such as the anterior cingulate cortex, thalamus, amygdala and ventral striatum amongst depressed PD patients compared to non-depressed patients [23]; the loss of dopamine and noradrenaline innervation in these brain regions also associated specifically with PD depression. Higher levels of depressive symptoms amongst PD patients also correlated with greater serotonin transporter (5-HTT) binding in the median raphe nuclei and limbic regions [24], highlighting the link between reduced serotonergic neurotransmission and PD depression.

These findings suggest the dysregulation of monoamine neurotransmitter systems in the development of depression in PD, and α-Syn has been shown to regulate dopamine, serotonin and norepinephrine transporter synaptic availability through subcellular binding, reducing the frequency of intracellular trafficking and resulting in reduced neurotransmitter levels at the cell surface [25]. Increased α-Syn levels, therefore, may lead to the dysregulation of dopaminergic, serotonergic and norepinephrine pathways [26] which mediate symptoms of mood disorders including depression [27, 28]. This is in keeping with the pathological hypothesis of depression as a result of neural system dysfunction in brain regions of the amygdala, hippocampus and prefrontal cortex, which are modulated by monoamine neurotransmitter systems [29]. Our findings that PD carriers of longer Rep1 alleles display more depressive symptoms might be explained by the modulating effect of increased *SNCA* expression resulting in greater dysregulation of neurotransmitter systems.

Fatigue in PD has been linked to serotonergic dysfunction within the basal ganglia and associated brain regions; PD patients with fatigue had significantly reduced serotonin transporter availability (as measured by PET ligand ^11^C-DASB) in striatal and limbic regions compared to patients without fatigue, suggesting that PD fatigue is associated with reduced serotonergic function in these areas [30], providing a pathophysiological basis for the trend towards an association between Rep1 allele length and FSS scores seen in our study. However, given the fundamental difficulty in defining PD fatigue [31], elucidating its pathological basis is challenging and evidence for a serotonergic basis of PD fatigue remains limited and inconclusive.

A limitation of our study remains that individual in-depth assessments for psychosis, autonomic and sensory symptoms, were not included in the present study. These assessments will be included in future longitudinal studies.

In conclusion, our study highlights that polymorphic variation in Rep1 is associated with non-motor symptom burden, in PD patients at early stages of the disease. Further longitudinal studies to assess in more detail the effects of Rep1 on psychosis, autonomic and sensory symptoms as well as functional studies to evaluate the role of Rep1 in non-dopaminergic systems may unravel new therapeutic targets for non-motor symptoms in PD. Identifying genetic contributions to PD non-motor symptoms will aid in early identification and stratification of patients at higher risk for non-motor symptoms, which will facilitate therapeutic monitoring and understanding of PD pathophysiology.

## MATERIALS AND METHODS

### Clinical recruitment and assessments

Subjects were recruited under the Early Parkinson’s Disease Longitudinal Singapore Study (PALS) between 2014 and 2019. All subjects fulfilled the National Institute of Neurological Disorders and Stroke (NINDS) criteria for a diagnosis of PD [32]. PD diagnosis had to be made less than a year before recruitment, with motor symptom onset less than two years prior to diagnosis. Patients with a history of significant neurological or psychiatric conditions were excluded. No familial PD patients were included; however, there were 9 patients who reported a history of PD in a first-degree relative, and 2 patients who reported a history of PD in a second-degree relative. Ethics approval was obtained from the relevant institutional review committee, and all subjects provided informed consent.

Functional status was determined using the Hoehn & Yahr (H&Y) rating scale [33] and motor symptom severity using the Movement Disorder Society Unified Parkinson’s Disease Rating Scale (MDS-UPDRS) Part III motor component [34]. Cognitive function was assessed using the Montreal Cognitive Assessment (MoCA) [35]. Where dopaminergic therapy had begun, the dosage was calculated and reported as cumulative levodopa equivalent daily dose (LEDD).

Overall non-motor symptom burden was screened for using the total score for the Non-Motor Symptoms Scale (NMSS) [36], which comprises nine domains each assessing a non-motor feature. Additionally, anxiety and depression were assessed specifically using the Hospital Anxiety and Depression Scale (HADS) [37], while levels of apathy and fatigue were evaluated via the Apathy Scale [38] and Fatigue Severity Scale (FSS) [39]. Sleep dysfunction was assessed using both the Epworth Sleepiness Scale (ESS) [40] and Pittsburgh Sleep Quality Index (PSQI) [41]. Higher scores indicate greater non-motor symptom severity and burden.

### *SNCA* Rep1 Polymorphism Genotyping

Fragment length analysis of *SNCA* Rep1 was performed using polymerase chain reaction (PCR) according to previous methods [16]. Rep1 allele lengths were coded as described by Farrer and colleagues [10]. Subjects carrying allele lengths coded as 1 or 2 were classified as “short” carriers, while those carrying allele lengths coded as 3 were classified as “long” carriers.

### Statistical analysis

Demographic and clinical characteristics were compared between long and short Rep1 groups using the Mann-Whitney U test for continuous variables, and chi-squared test for categorical variables. Multivariable linear regressions were performed to investigate the association between Rep1 allele length and non-motor outcomes, adjusting for statistical and clinical confounders (age, sex, disease duration, MoCA score and LEDD). Significance level was set at *p* < 0.05, with adjustments made using Bonferroni correction for multiple testing. Statistical analysis was performed using IBM SPSS Statistics, version 21.0.
